# Emergency admission, previous delirium tremens and increased liver stiffness as risk factors for severe alcohol withdrawal – A prospective study

**DOI:** 10.1371/journal.pone.0320083

**Published:** 2025-03-19

**Authors:** Tobias Zellner, Jan-Christopher Metzger, Elias Bekka, Matteo Rabaioli, Konrad Stock, Minh-Truc Vo-Cong, Sabrina Schmoll, Eva-Carina Heier, Florian Eyer, Raphael Stich

**Affiliations:** 1 Division of Clinical Toxicology and Poison Centre Munich, Department of Internal Medicine II, TUM School of Medicine and Health, TUM University Hospital, Technical University of Munich, Munich, Germany; 2 Clinical Pharmacology and Toxicology, Department of General Internal Medicine, Inselspital, Bern University Hospital, University of Bern, Bern, Switzerland; 3 Division of Nephrology, Ultrasound Section, Department of Internal Medicine II, TUM School of Medicine and Health, TUM University Hospital, Technical University of Munich, Munich, Germany; Universitatsklinikum Leipzig, GERMANY

## Abstract

**Objective:**

To determine if increased liver stiffness (ILS) is a risk factor for patients with alcohol withdrawal to develop severe alcohol withdrawal symptoms (SAWS) like delirium tremens (DT) or withdrawal seizures (WS).

**Method:**

Prospective inclusion of 394 patients undergoing withdrawal treatment between 2013-2021. Laboratory exams, history, physical examination, abdominal sonography with elastography and FibroScan® measurements were performed. Primary endpoint was SAWS defined as DT and/or WS. Patients with >  12.5 kPa stiffness in FibroScan® and >  1.75 m/s in Acoustic Radiation Force Impulse Imaging were considered ILS, patients with both measurements below the respective cut-off were ILS negative. Univariate analysis with receiver operating characteristic curve analysis and multivariate analysis were performed.

**Results:**

78 patients (19.8%) had ILS. Of these, 28 patients developed complications despite treatment. SAWS correlated significantly with patients with ILS. Further significant correlations were emergency hospital admission, Alcohol Withdrawal Scale ≥  5, lower potassium, elevated bilirubin, increased Gamma-GT, thrombocytopenia, previous WS, and previous DT. In multivariate binary regression analysis, odds ratio for SAWS was 5.4 for emergency admission, 3.5 for previous DT and 2.2 for ILS, even if the significance level for the last parameter was missed.

**Conclusions:**

Patients with ILS have an increased risk of developing SAWS, as well as patients with emergency admission and previous DT among other markers. Treatment in an appropriately equipped facility is recommended for patients with this risk profile which can be measured easily by a general practitioner or in an emergency department.

## Introduction

Harmful use of alcohol is a significant health risk all over the world. Around 50% of all cases of liver cirrhosis (LC) are due to alcohol abuse [1,2]. Westman et al. reported that patients with alcohol use disorder (AUD) in Denmark, Finland and Sweden have a life expectancy that is 24–28 years lower compared to the general population [3]. Physicians should therefore be encouraged to actively query alcohol consumption and focus on achieving alcohol abstinence in patients with AUD [4]. This not only prevents acute alcohol-related complications such as accidents, but is also a prerequisite for liver regeneration [5].

During alcohol detoxification the brain enters a state of hyperexcitability which may result in alcohol withdrawal syndrome (AWS) with autonomic symptoms like sweating, tremors, anxiety, insomnia, tachycardia, and hypertension. Between less than 1% of general population to around 50% of patients with AUD develop AWS, 5–20% of those develop severe alcohol withdrawal syndrome (SAWS) which can cause life threatening complications like delirium tremens (DT) and withdrawal seizures (WS) [6,7]. Identifying beforehand which patient will develop AWS or SAWS is a great challenge. Therefore, various tools like the “Prediction of Alcohol Withdrawal Severity Scale” (PAWSS) have been developed [8].

Despite the enormous number of patients with AUD worldwide, no standardized procedure for carrying out alcohol detoxification has been established [9]. Most patients – especially with comorbidities or a history of AWS – are treated in an inpatient setting, and benzodiazepines are the most commonly used drug for detoxification [10]. However, there is no clear recommendation on how to perform alcohol detoxification in patients with LC. Furthermore, it remains unclear if patients with LC have an increased risk for developing SAWS due to insufficient and inconsistent data. Increased liver stiffness (ILS) comprising alcoholic liver cirrhosis (ALC), fibrosis, alcoholic steatohepatitis (AS), liver disease, or liver injury can be measured easily via FibroScan® [11,12] or Acoustic Radiation Force Impulse Imaging (ARFI) [13] by a general practitioner (GP) or in the emergency department (ED), if equipped with suitable devices. Therefore, we focused on patients with ILS to determine their risk of complications during inpatient alcohol withdrawal treatment (AWT).

## Methods

### Study design

This study was conducted in accordance with the principles of the Declaration of Helsinki and Good Clinical Practice guidelines. Ethical approval was obtained by our ethics commission (5743/13). Written informed consent was obtained from all patients before inclusion in the study. The study started on 1^st^ October 2013 and is ongoing. Data collection for this paper was between 1^st^ October 2013 and 31^st^ November 2021. The authors had access to information that could identify individual participants during or after data collection.

**Fig 1:**
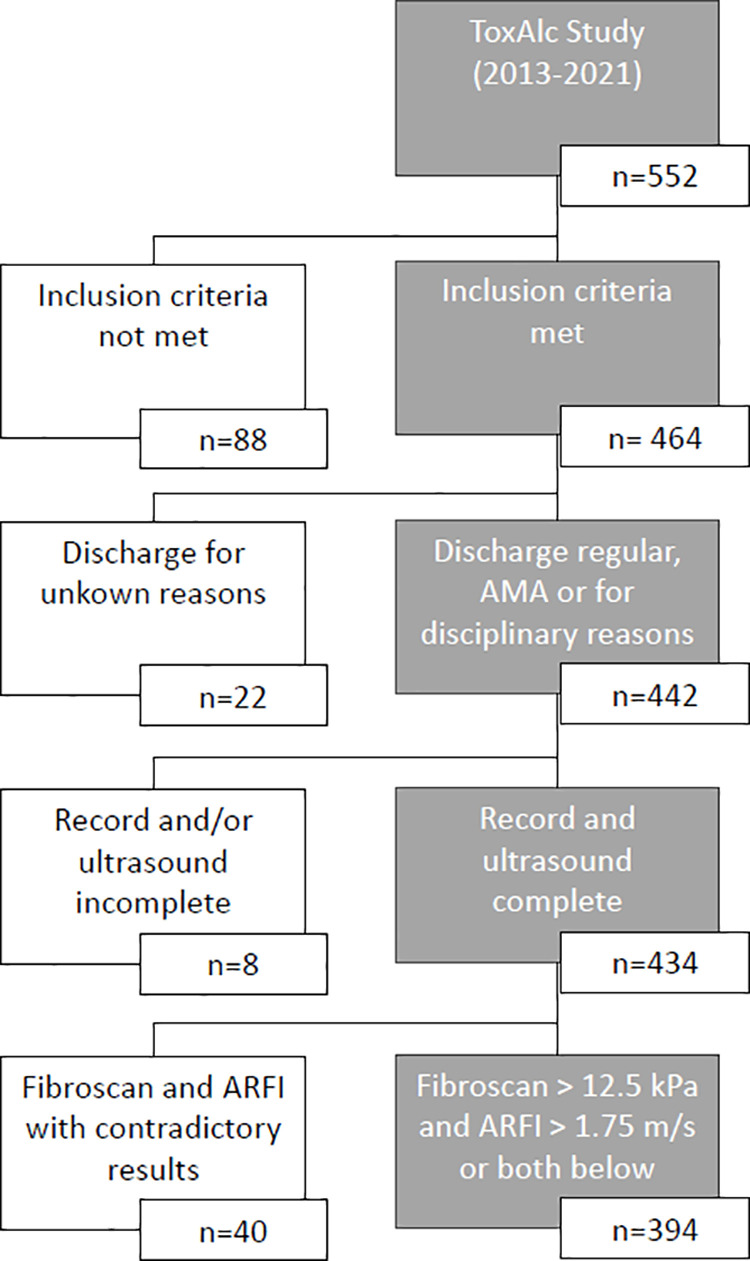
Enrollment diagram.

Our department is specialized in detoxification of alcohol and/or other recreational drugs and treatment of acute intoxications with an attached medical intermediate care unit and intensive care unit (ICU). We prospectively included patients with AUD admitted for AWT electively or via the ED with alcohol intoxication and subsequent AWS, receiving AWT in our department. AWS was treated with either clomethiazole or benzodiazepines and/or clonidine, for anticonvulsive prophylaxis valproic acid or levetiracetam was administered. Patients with regular consumption of other illegal drugs, missing consent or younger than 18 years were excluded. Patients who refused further treatment due to unknown reasons were also excluded. Patients with a discharge against medical advice (AMA) or for disciplinary reasons were included, provided consent was not revoked and data collection was complete. Enrollment can be seen in Fig 1.

Medical and social history were documented using standardized questionnaires including drinking habits, alcohol intake, amount and duration of alcohol consumption, drinking type according to Jellinek [14] and the length of abstinence before starting withdrawal treatment. Patients needed to fulfill the ICD-10 criteria for alcohol use disorder. Physical examination, laboratory exams and abdominal ultrasound were conducted according to a standardized protocol. Acute viral hepatitis, autoimmune hepatitis, hemochromatosis and Morbus Wilson were ruled out by laboratory tests.

Abdominal ultrasound (Siemens Acuson S2000 and Siemens Acuson Healthineers Sequoia) was conducted by two DEGUM-certified (German Society for Ultrasound in Medicine) examiners in the context of this study. Acoustic Radiation Force Impulse Imaging (ARFI) was measured in m/s in liver segment VIII. Each patient received FibroScan® measurements which were measured in kPa (Echosense FibroScan® touch 502, software version C3.2). Fibroscan® examinations were carried out in gastroenterology and ARFI measurements in our nephrology department.

ILS was diagnosed using elastography with an ARFI >  1.75 m/s and FibroScan® with a stiffness >  12.5 kPa [12,13,15]. Patients with both measurements below this threshold were considered ILS negative. Patients with contradictory measurements (ARFI <  1.75 m/s and stiffness >  12.5 kPa or ARFI >  1.75 m/s and stiffness <  12.5 kPa) were excluded from the study (n = 40).

Primary endpoint was the occurrence of SAWS. This was defined as AW with complications like DT and/or WS. DT was diagnosed clinically in patients developing disorientation, hallucinations, confusion and/or agitation with the need for medical intervention. Hepatic encephalopathy had to be ruled out by clinical (asterixis), radiological (cranial computed tomography or magnetic resonance imaging studies) and/or laboratory examinations (increased ammonia level). WS were defined as grand mal epileptic seizures during AWT in the absence of structural epilepsy or other etiologies.

All data was entered into the database and checked for consistency, plausibility and entry errors by the same supervising senior physician.

### Statistical analysis

All data was entered in a predefined database (Claris FileMaker Pro®, Version 20.3.2.201, Claris International, Sunnyvale, California, USA). Statistical analysis was performed using IBM SPSS V.28 (IBM, Armonk, New York, USA). Qualitative variables were summarized using absolute numbers and percentages. Qualitative variables were tested for normal distribution using the Kolmogorov-Smirnov-Test and checked graphically and displayed as mean plus standard deviation (SD) for normally distributed variables. For not normally distributed variables, median plus range are displayed. The Chi-square test was applied for qualitative variables, the Wilcoxon-Mann-Whitney-U-Test for not normally distributed quantitative variables and the t-Test for normally distributed quantitative variables.

Continuous predictive variables were analyzed with receiver operating characteristic (ROC) curves and cut-offs were tested at the 90% specificity and sensitivity thresholds and at the highest Youden’s index.

The null hypothesis was that patients with ILS have a similar risk of SAWS as patients without ILS. This hypothesis was tested using the Chi-square test. A p value <  0.05 was considered statistically significant. Power calculation with a beta-error of 80% was performed and 382 patients were required to power the study.

Secondary factors associated with ILS and SAWS were also analyzed and a p value <  0.05 was considered a statistically significant correlation. Multivariate binary logistic regression analysis was then performed for three parameters to calculate odds ratios (ORs) and their respective 95% confidence interval (CI). E-values were calculated according to VanderWeele et al [16].

This analysis was not pre-registered and therefore the results should be considered exploratory.

## Results

### Baseline characteristics

A total of 394 patients were included (see Fig 1). The mean age was 50 years, with a male predominance. 173 patients were smokers, the mean body mass index (BMI) was 25. Patients all fulfilled the ICD-10 criteria for alcohol addiction and self-reported a median consumption of 176 g ethanol per day. 18 patients underwent detoxification for the first time – the remaining patients already had undergone a median of three detoxifications. 164 were admitted to the hospital as an emergency. 99 patients reported previous WS and 60 previous DT. The detoxification was completed in 366 patients, discontinued due to disciplinary reasons in six and discontinued because patients left treatment AMA in 22 cases.

Of the 394 patients included, 78 patients were diagnosed with ALC/AS. The differences between these groups are shown in Table 1.

**Table 1:** Normal liver stiffness vs. increased liver stiffness.

Overall (n = 394)	NLS (n = 316)		ILS (n = 78)		P value
**Sex**					0.377
Male	219	69.3%	50	64.1%	
Female	97	30.7%	28	35.9%	
**Age** (mean ± SD)	49.0	±11.5	52.2	±10.4	**0.024**
**BMI** (mean ± SD)	24.5	±4.4	27.0	±7.1	**<0.001**
**SAWS**					**0.045**
No (n = 366)	298	94.3%	68	87.2%	
Yes (n = 28)	18	5.7%	10	12.8%	
**Smoker**					0.287
Yes	140	44.3%	33	42.3%	
No	20	6.3%	9	11.5%	
Unknown	156	49.4%	36	46.2%	
**AWS**					0.126
< 5	102	32.8%	32	42.1%	
> / = 5	209	67.2%	44	57.9%	
**Delirium tremens in history**					0.693
No	269	85.1%	65	83.3%	
Yes	47	14.9%	13	16.7%	
**Withdrawal seizure in history**					0.683
No	238	75.3%	57	73.1%	
Yes	78	24.7%	21	26.9%	
**Ethanol consumption in g/d**	176.0	9.5–1094	176.0	11.9–480	0.763
**Drinking type**					0.632
Alpha (n = 28)	20	6.6%	8	10.7%	
Beta (n = 10)	9	3%	1	1.3%	
Gamma (n = 124)	98	32.5%	26	34.7%	
Delta (n = 152)	122	40.4%	30	40%	
Epsilon (n = 51)	44	14.6%	7	9.3%	
Mixed (n = 12)	9	3%	3	4%	
**Elective admission**					**0.045**
No	124	39.4%	40	51.9%	
Yes	191	60.6%	37	48.1%	
**Medication for AWS**					0.441
No	72	22.8%	21	26.9%	
Yes	244	77.2%	57	73.1%	
**Anticonvulsant therapy**					0.070
No	10	3.2%	6	7.7%	
Yes	306	96.6%	72	92.3%	
**Admission to ICU**					0.425
No (n = 435, 93.7%)	299	94.6%	72	92.3%	
Yes	17	5.4%	6	7.7%	
**Discharge from hospital**					0.172
Disciplinary	6	1.9%	0	0.0%	
Regular	295	93.4%	71	91.0%	
AMA	15	4.7%	7	9.0%	
**AWS score at admission**	0	0–12	0	0–10	0.951
**AWS score maximum**	5	0–17	5	0–16	0.899
**PAWSS**	5	0–9	5	1–8	0.803
**Stiffness in kPa**	5.3	2.4–12	39.7	12.5–75	**<0.001**
**ARFI in Segment VIII in m/s**	1.19	0.70–1.75	2.60	1.75–4.26	**<0.001**

Counts with %, mean with standard deviation, and median with range.

Abbreviations: NLS=Normal liver stiffness; ILS =  Increased liver stiffness; BMI=Body mass index; AWS=Alcohol withdrawal scale; PAWSS = Prediction of Alcohol Withdrawal Severity Scale; ARFI = Acoustic Radiation Force Impulse Imaging.

A total of 301 patients developed an AWS that required withdrawal specific medication: 262 patients received clomethiazole, 39 lorazepam, and 107 clonidine. 378 patients received prophylactic anticonvulsant therapy: 227 patients received valproic acid, 137 patients were treated with levetiracetam, eight patients received a combination of valproic acid and levetiracetam, and six patients received carbamazepine in addition to valproic acid. There was no significant correlation between the type of anticonvulsant therapy and SAWS (p = 0.181). Nevertheless, 28 patients developed SAWS (WS n = 4, DT n = 22, WS +  DT n = 2). Of these, 23 patients had to be treated on our ICU due to increased need for sedation, fixation, neuroleptic treatment or intubation due to sedation or aspiration pneumonia.

### Normal liver stiffness (NLS) vs. ILS

The differences for patients with NLS vs. ILS are shown in Table 1. In the ILS group there was one patient with WS, eight patients with DT and one with both (overall ten patients with SAWS, 12.8%). In the NLS group, the numbers were three, 14 and one, respectively (18 patients with SAWS, 5.8%). In the ILS group, patients were older (p = 0.024) and had a higher BMI (p=<0.001). Patients with ILS had a higher rate of emergency hospital admissions (p = 0.045). There was no difference in alcohol consumption or drinking type.

The differences in laboratory exams in the NLS vs. ILS group are shown in Table 2. As expected, patients with ILS had thrombocytopenia, lower hemoglobin, increased Gamma-GT, AST and bilirubin and lower Quick/higher INR. They also showed lower potassium levels and increased MCV at admission.

**Table 2:** Laboratory examination results on admission normal liver stiffness vs. increased liver stiffness.

Overall (n = 394)	NLS (n = 316)	SD ±	ILS (n = 78)	SD ±	P value
**Sodium** (mmol/l)	138	6	137	6	0.186
**Potassium** (mmol/l)	4.4	0.5	4.0	0.5	**<0.001**
**Creatinine** (mg/dl)	0.8	0.2	0.7	0.3	0.084
**GFR** (ml/min)	60	3	59	4	0.520
**BUN** (mg/dl)	11	9	10	11	0.359
**Thrombocytes** (G/l)	221	97	125	80	**<0.001**
**Hgb** (g/dl)	14.6	1.8	12.8	2.3	**<0.001**
**MCV** (fl)	93	6	97	7	**<0.001**
**GGT** (U/l)	393	775	854	634	**<0.001**
**AST** (U/l)	96	110	160	115	**<0.001**
**ALT** (U/l)	78	94	68	44	0.163
**Bilirubin** (mg/dl)	1.0	0.6	1.3	0.2	**<0.001**
**INR**	1.0	0.6	1.3	0.2	**<0.001**
**Quick** (%)	101	12	68	18	**<0.001**
**Blood ethanol level** (g/l)	1.77	1.50	1.49	1.37	0.132

Mean with standard deviation.

Abbreviations: NLS=Normal liver stiffness; Hgb =  Hemoglobin; MCV =  Mean corpuscular hemoglobin; GGT =  Gamma-glutamyl Transferase; AST =  Aspartate aminotransferase; ALT =  Alanine Aminotransferase.

### No-SAWS vs. SAWS

The differences for the patients with and without SAWS are shown in Table 3. The risk for developing SAWS was significantly higher in patients with ILS (p = 0.028). Further factors correlating significantly with SAWS were older age (p = 0.035), AWSS ≥  5 (p = 0.006), admission to hospital as emergency (p < 0.001), as well as lower potassium (p < 0.001), elevated bilirubin (p=<0.001), increased Gamma-GT (p = 0.019) and AST (p = 0.003), increased MCV (p = 0.002), and thrombocytopenia (p < 0.001). The differences in laboratory exams in the no-SAWS vs. SAWS groups are shown in Table 4. Furthermore, a history of WS and DT increased the risk for SAWS significantly (p = 0.025 and p = 0.002), as well as a higher maximal AWS score (p < 0.001). The PAWSS did not significantly correlate with SAWS. There was no difference in alcohol consumption or drinking type.

**Table 3:** No severe alcohol withdrawal syndrome vs. severe alcohol withdrawal syndrome.

Overall (n = 394)	No-SAWS (n = 366)		SAWS (n = 28)		P value
**Sex**					0.638
Male	251	68.6%	18	64.3%	
Female	115	31.4%	10	35.7%	
**Age** (mean ± SD)	49.3	11.4	54.0	9.0	**0.035**
**BMI** (mean ± SD)	25.0	5.2	24.3	4.1	0.486
**Smoker**					0.335
Yes	161	44.0%	12	42.9%	
No	25	6.8%	4	14.3%	
- Unknown	180	49.2%	12	42.9%	
**AWS**					**0.006**
< 5	131	36.5%	3	10.7%	
> / = 5	228	63.5%	25	89.3%	
**Delirium tremens in history**					**0.002**
No	316	86.3%	18	64.3%	
Yes	50	13.7%	10	35.7%	
**Withdrawal seizure in history**					**0.025**
No	279	76.2%	16	57.1%	
Yes	87	23.8%	12	42.9%	
**Ethanol consumption** in g/d	198.5	147.2	174.8	96.9	0.959
**Drinking type**					0.811
Alpha (n = 28)	26	7.4%	2	7.4%	
Beta (n = 10)	10	2.9%	0	0%	
Gamma (n = 124)	115	32.9%	9	33.3%	
Delta (n = 152)	139	39.7%	13	48.1%	
Epsilon (n = 51)	48	13.7%	3	11.1%	
Mixed (n = 12)	12	3.4%	0	0%	
**Elective admission**					**<0.001**
No	142	39.0%	22	78.6%	
Yes	222	61.0%	6	21.4%	
**Medication for AWS**					0.096
No	90	24.6%	3	10.7%	
Yes	276	75.4%	25	89.3%	
**Anticonvulsant therapy**					0.892
No	15	4.1%	1	3.6%	
Yes	351	95.9%	27	96.4%	
**Admission to ICU**					**<0.001**
No (n = 435, 93.7%)	350	95.6%	21	75.0%	
Yes	16	4.4%	7	25.0%	
**Discharge from hospital**					0.316
Disciplinary	6	1.6%	0	0.0%	
Regular	338	92.3%	28	100.0%	
AMA	22	6.0%	0	0.0%	
**AWS score at admission**	0	0–12	3	0–9	0.089
**AWS score maximum**	5	0–13	8	0–17	**<0.001**
**PAWSS**	5	0–9	5	2–8	0.717
**Stiffness in kPa**	5.7	2.4–75.0	8.2	3.5–65.5	**0.038**
**ARFI in Segment VIII in m/s**	1.24	0.70–4.26	1.45	0.92–3.45	0.100
**Liver stiffness**					**0.028**
Normal	298	81.4%	18	64.3%	
Increased	68	18.6%	10	35.7%	

Counts with %, mean with standard deviation, and median with range.

Abbreviations: BMI=Body mass index; AWS=Alcohol withdrawal scale; PAWSS = Prediction of Alcohol Withdrawal Severity Scale.

**Table 4:** Laboratory examination results on admission no severe alcohol withdrawal syndrome vs. severe alcohol withdrawal syndrome.

Overall (n = 394)	No-SAWS (n = 366)	SD ±	SAWS (n = 28)	SD ±	P value
**Sodium** (mmol/l)	138	6	137	6	0.138
**Potassium** (mmol/l)	4.3	0.5	3.9	0.4	**<0.001**
**Creatinine** (mg/dl)	0.8	0.2	0.7	0.2	0.123
**GFR** (ml/min)	60	3	60	0	0.466
**BUN** (mg/dl)	11	10	9	8	0.461
**Thrombocytes** (G/l)	210	100	106	49	**<0.001**
**Hgb** (g/dl)	14.3	2.0	13.7	2.0	0.144
**MCV** (fl)	94	7	98	6	**0.002**
**GGT** (U/l)	446	729	971	1095	**0.019**
**AST** (U/l)	104	110	171	144	**0.003**
**ALT** (U/l)	76	89	82	51	0.711
**Bilirubin** (mg/dl)	1.1	1.6	2.4	2.7	**<0.001**
**INR**	1.1	0.6	1.1	0.2	0.507
**Quick** (%)	96	18	86	22	**0.013**
**Blood ethanol level** (g/l)	1.71	1.46	1.81	1.67	0.748

Mean with standard deviation.

Abbreviations: Hgb =  Hemoglobin; MCV =  Mean corpuscular hemoglobin; GGT =  Gamma-glutamyl Transferase; AST =  Aspartate aminotransferase; ALT =  Alanine Aminotransferase.

Patients with SAWS had a significantly increased risk of requiring ICU care (p < 0.001). However, there was no significant difference in discharge (all patients were discharged alive, in the SAWS group all regularly).

**Fig 2:**
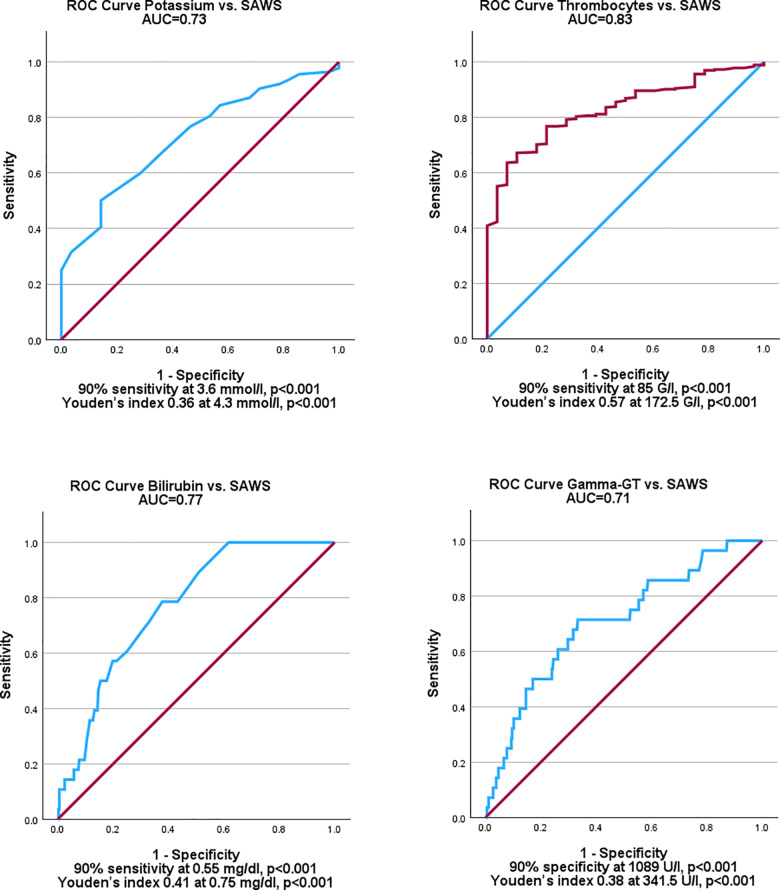
Receiver operator characteristic curve analysis of potassium, thrombocytes, bilirubin, and Gamma-GT.

ROC curves had a favorable area under the curve (AUC) for continuous variables which correlated significantly with SAWS (see Fig 2). Several cut-offs could be established either at the 90% sensitivity, the 90% specificity threshold or at the highest Youden’s index: lower potassium (p < 0.001; AUC = 0.73; cut-off of with 90% sensitivity at 3.6 mmol/l, p < 0.001; cut-off with a Youden’s index of 0.36 at 4.3 mmol/l, p < 0.001), elevated bilirubin (p < 0.001; AUC = 0.77; cut-off of with 90% sensitivity at 0.55 mg/dl, p < 0.001; cut-off with a Youden’s index of 0.41 at 0.75 mg/dl, p < 0.001), increased Gamma-GT (p < 0.001; AUC = 0.71; cut-off with 90% specificity at 1089 U/l, p < 0.001; cut-off with a Youden’s index of 0.38 at 341.5 U/l, p < 0.001), thrombocytopenia (p < 0.001; AUC = 0.83; cut-off of with 90% sensitivity at 85 G/l, p < 0.001; cut-off with a Youden’s index of 0.57 at 172.5 G/l, p < 0.001) and increased stiffness (p = 0.026; AUC = 0.64; no significant cut-off at 90% specificity and sensitivity thresholds for SAWS, cut-off with a Youden’s index of 0.26 at 6.05 kPa, p = 0.017).

In multivariate binary regression analysis (Table 5), emergency admission increased the risk for SAWS with an odds ratio (OR) of 5.4 (95% confidence interval (CI) 2.2–13.9), previous DT increased the risk with an OR of 3.5 (95% CI 1.5–8.3) and ILS increased the risk with an OR of 2.2 (95% CI 0.9–5.2). The calculated E-values for emergency admission were 10.4 for OR and 3.82 for its CI, for previous DT 6.46 for OR and 2.37 for its CI, and for ILS 3.82 for OR and 1 for its CI.

**Table 5:** Multivariate binary logistic regression analysis.

Predictor variable	Regression coefficient	Standard error	Wald χ2	*p*	Odds ratio (95% CI)
Increased stiffness	0.784	0.436	3.225	0.073	2.2 (0.9–5.2)
Previous delirium	1.255	0.442	8.051	0.005	3.5 (1.5–8.3)
Emergency admission	1.692	0.480	12.441	<0.001	5.4 (2.1–13.9)

## Discussion

We report data from a prospectively collected cohort undergoing inpatient alcohol detoxification. Our focus was on risk factors for the occurrence of SAWS, particularly in patients with ILS. We were able to identify admission via ED, previous DT and ILS as risk factors for SAWS as well as AWS ≥  5, hypokalemia, elevated bilirubin, increased Gamma-GT, and thrombocytopenia.

In literature, SAWS has no distinct definition although most authors define it as AWS with occurrence of DT and/or WS [17]. A crucial point in conducting detoxification is predicting which patient will develop AWS or SAWS and will therefore need medical or even ICU treatment. Well-established prognostic factors for complications such as previous DT or WS during AWS, derived from the patient’s history, are reported in literature [17–21]. The PAWSS, although initially evaluated in an unselected group of patients, proved in a meta-analysis to be the best score to predict AWS across studies [21,22]. In our pre-selected group of patients, there was no correlation between PAWSS and the occurrence of SAWS. This could be expected, since the PAWSS was not designed to predict SAWS but AWS. An overactivity of the autonomic nervous system, such as tachycardia, sweating, tremor, nausea and increased temperature, represented in the AWS ≥  5 was also associated with DT in several studies [18,20]. Especially patients with DT and/or WS in their medical history are at high risk of experiencing SAWS, often requiring ICU treatment [23]. Recently, Denk et al. showed that liver disease is a risk factor for delirium in the ICU, as seen in the ILS, elevated bilirubin, increased Gamma-GT, and thrombocytopenia in our study [24]. They also emphasized that delirium and hepatic encephalopathy are distinct entities though clinical differentiation can be difficult. This could be the reason for the significant association of ILS and SAWS in our study, since patients with liver disease usually have ILS [25]. Hildenbrand et al. observed that the risk for delirium was significantly increased in patients with LC or acute renal failure [26]. Other risk factors like hypokalemia and thrombocytopenia have shown significant prognostic value in various studies – which is also in line with our findings [27–29]. Alkhachroum et al. performed a large retrospective cohort study and found that LC was associated with status epilepticus [30]. Although no clear pathomechanism linking SAWS to ILS has been described, generally accepted measures to reduce the risk for delirium such as early mobilization, orientation, sleep protocols, noise reduction, familiarity, cognitive stimulation, and pain management can be employed. Patients with risk factors might require more aggressive pharmacological treatment and possibly early transfer to a facility with an in-house ICU. Prophylactic anticonvulsive medication might also reduce the risk of SAWS.

Fibroscan® or ARFI measurements could be an easily applicable tool for GPs or in the ED to create a risk stratification and send patients with AUD for AWT to a specialized unit. Currently, few GPs or EDs are equipped with Fibroscan® or ARFI measuring ultrasound machines, however, this could change in the future as the market for these devices is growing and costs are likely to fall. However, it is important to perform elastography with certain criteria to get good results: All measurements were taken at the end of expiration. The patients were examined mostly in the morning, all on an empty stomach using both elastography methods in a blinded manner by different examiners in different departments. No patient had immediate postprandial symptoms; this was also evident in sonography. We examined different measurement protocols in different liver segments for all patients, with the measurement in S VIII yielding the most reproducible and consistent values. We carried out all measurements vertically at a depth of two centimeters into liver tissue, avoiding larger vessels. When examining liver segment VIII, we took 10–15 measurements each and recorded the mean, median and standard deviation.

The time at which the ILS was measured also seems to be decisive: the transaminases usually fall relatively quickly after the onset of AWS, often after about six days [31]. Prior studies have shown decreasing liver stiffness during the first week of alcohol detoxification [31]. This can probably be recorded with Fibroscan® measurements with a reduced stiffness. In the AWS phase, transaminases are not yet expected to fall and therefore the ILS will still be high at this time. AST and GGT were significantly higher in the ILS patients, as well as in the SAWS patients. At an examination after 1–2 weeks, for example, the ILS could already have fallen by the time the laboratory improves [31].

Barrio et al. showed that the risk for SAWS was significantly lower in patients with diagnosed ALC with a daily intake of > 80 g/d ethanol [32]. Other retrospective studies showed no significant correlation between ALC and development of DT or WS [20,27,33]. Besides, there are not many other factors that have demonstrated a robust predictive value across studies.

Due to the combination of a detoxification, intermediate care and intensive care unit, the proportion of somatically more severely affected patients is high in our cohort, reflected by the ratio of nearly 20% of patients presenting with ILS. Our rate of severe complications of 7.1% is consistent with other studies [7,18,22,29,34]. Noteworthy, we observed a lower rate of WS and a higher percentage of DT, especially in the ILS-group. The low number of WS is probably attributable to our prophylactic anticonvulsant therapy.

Noteworthy, none of our patients died. Other studies found higher mortality rates [35,36].

## Limitations

This is a monocentric study, so hospital or region-specific factors like choice of medication are likely. Furthermore, not all patients meeting inclusion criteria were included in our study either due to missing consent, due to organizational circumstances or due to contradictory ultrasound results, which could have created a selection bias. We decided to exclude patients with contradictory results as studies have shown that after a recent alcohol consumption there is a risk to overestimate stage of fibrosis [37].

Liver biopsy is the gold standard for diagnosing ALC/AS. However, with developments in sonography such an invasive procedure should be limited to patients where diagnosis remains unclear [38]. It has been shown that FibroScan® and ARFI are valid diagnostic tools [13,15]. For FibroScan®, different cut-offs have been suggested for patients with AUD due to the increased prevalence of AS: For patients without AUD, 12.5 kPa is the established cut-off for cirrhosis. For patients with AUD higher cut-offs like 21.2 kPa have been suggested [11,12,15]. Nevertheless, we used 12.5 kPa as cut-off, since measurements below 12.5 kPa have a negative predictive value of 92% and 93% for ruling out severe fibrosis and cirrhosis [12]. This is however the reason why we cannot differentiate between liver diseases without biopsy.

There may be a selection error by excluding patients with contradictory measurements (ARFI <  1.75 m/s and stiffness >  12.5 kPa or ARFI >  1.75 m/s and stiffness <  12.5 kPa) from our study (n = 40). However, this exclusion helps ensure that the patients enrolled in the study can clearly be assigned into their respective groups. Due to the small size of patients in the SAWS group, only three variables were tested in multivariate binary regression analysis. A larger study would be necessary to test for more confounders.

The fact that we are a medical department specialized in detoxification could result in patients who are different to those in other detoxification settings, e.g., psychiatric hospitals, with reduced comparability. Alcohol intake was self-reported and therefore hard to corroborate.

Levetiracetam has become the standard in our department for every alcohol detoxification since July 2017. Alternatively, valproic acid was used for patients without LC as the standard regimen before July 2017. In 2017 a “Dear Doctor Letter” was issued against Valproic acid in Germany, necessitating the switch to levetiracetam. The change in anticonvulsant therapy during the study period may have influenced the occurrence of SAWS, but no significant association between anticonvulsant therapy and SAWS was found in our cohort.

## Conclusion

In our prospective study we found that patients with ILS had a significantly increased risk to develop SAWS, defined as WS and/or DT. In our cohort, the PAWSS did not significantly correlate with the occurrence of SAWS. Factors correlating with SAWS were emergency admission, previous DT, hypokalemia, thrombocytopenia, hyperbilirubinemia, increased Gamma-GT, and a history of SAWS. In multivariate binary regression analysis, emergency admission (5.4), previous DT (OR 3.5) and ILS (OR 2.2, not significant) all increased the risk for developing SAWS, although the effect of an emergency admission was strongest. All these risk factors are associated with medical emergency problems and most risk factors are easily available at admission. ARFI or Fibroscan® measurements could be easily available for GPs or EDs in the future, to improve risk stratification for patients with AUD. We recommend treating patients planned for AWT with emergency admission, previous DT or ILS in an appropriately equipped medical facility with an in-house ICU. Future investigation should focus on developing clinically easily applicable scores which can already be recorded upon admission, to identify patients at risk for SAWS and to adjust or intensify proper treatment.

In summary, the exact pathomechanism for the increased risk in ILS patients for SAWS remains unknown. Therefore, future studies should focus on this to help understand the underlying processes and improve therapeutic concepts.

## Abbreviations

ALC: Alcoholic Liver Cirrhosis
AMA: Against Medical Advice
ARFI: Acoustic Radiation Force Impulse Imaging
AUC: Area Under the Curve
AUD: Alcohol Use Disorder
AS: Alcoholic Steatohepatitis
AW: Alcohol Withdrawal
AWS: Alcohol Withdrawal Syndrome
AWSS: Alcohol Withdrawal Severity Scale
AWT: Alcohol Withdrawal Treatment
BMI: Body Mass Index
CI: Confidence Interval
DT: Delirium Tremens
ED: Emergency Department
GP: General Practitioner
ICU: Intensive Care Unit
ILS: Increased Liver Stiffness
LC: Liver Cirrhosis
NLS: Normal Liver Stiffness
OR: Odds Ratio
ROC: Receiver Operating Characteristic
SAWS: Severe Alcohol Withdrawal Syndrome
SD: Standard Deviation
WS: Withdrawal Seizure.
